# Biliary leak embolization with conformable embolic

**DOI:** 10.1186/s42155-026-00733-4

**Published:** 2026-07-15

**Authors:** Abdul Khan, Nicholas Xiao

**Affiliations:** 1 University of Chicago, 5841 S. Maryland Avenue, MC2026, P220., Chicago, IL 60637 USA; 2 Midwest Radiology, Roseville, MN USA

## Abstract

Biliary leaks refractory to initial endoscopic or percutaneous decompressive drainage are commonly difficult to definitively manage. This case series describes the use of Obsidio Conformable Embolic (Boston Scientific, Marlborough, MA, USA) for successful embolization of refractory biliary leaks, highlighting it as a promising tool for managing this challenging patient population.

## Introduction

The majority of biliary leaks can be managed with endoscopic drainage or percutaneous drain placement. Biliary leaks refractory to initial management may require operative repair; however, surgical intervention is often associated with greater morbidity. Embolization of biliary leaks has been previously described with the use of n-BCA glue and coils; however, in the author’s experience, these agents are only effective in a fraction of cases [1]. Obsidio is a conformable embolic made from hydrogel with unique shear-thinning properties which allows for various embolic effects depending on injection forces employed by the operator. This case series illustrates the use of this conformable embolic for definitive treatment of previously refractory biliary leaks.

## Case 1

A 63-year-old man whom underwent robotic-assisted laparoscopic cholecystectomy was subsequently found to have a bile leak and biloma on hepatobiliary iminodiacetic acid (HIDA) imaging. A percutaneous drain was placed into the biloma. Despite six endoscopic retrograde cholangiopancreatography (ERCP) interventions and multiple endoscopic stents, the patient’s biloma persisted for over 6 months.

**Fig. 1:**
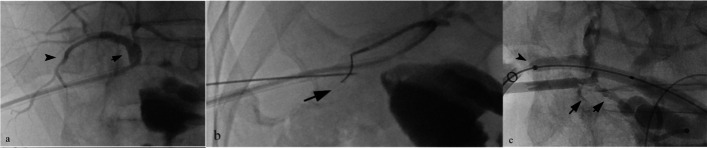
**a** Catheterization of a peripheral intrahepatic duct (arrowhead) through the biloma cavity (arrow). **b** Percutaenous puncture of peripheral hepatic duct with aid of a snare. **c** Embolization of the leak carried out with Obsidio embolic (arrow). A balloon was inflated at the time of embolization to prevent nontarget embolization of intrahepatic ducts (arrowhead)

Careful review of imaging suggested a duct of Lushka transection. Using an angled catheter, a glidewire was passed from the percutaneous biloma drain access through the defect in the duct of Lushka and into a contiguous peripheral intrahepatic duct (Fig. 1a). A snare was placed through the catheter in the peripheral duct. A second percutaneous access was achieved through the snare, and a wire was passed through the new transhepatic access with establishment of a through-and-through wire exiting through the biloma cavity access (Fig. 1b). A sheath was placed trans-hepatically and a second wire was passed through the common bile duct and into the small bowel. A balloon was placed in the intrahepatic duct across the biliary leak from the new transhepatic access to prevent intra-hepatic ductal delivery of embolic (Fig. 1c). A microcatheter was then passed over the through-and-through wire from the biloma-side. The through-wire was removed. Obsidio embolization was then performed through the microcatheter using fast, forceful injection to achieve a larger conformable plug trailing through the leaking duct of Lushka and into the biloma cavity. The entire 1 cc syringe was used. A new 8.5-F internal–external biliary drain and the biloma drain were replaced.

The patient’s biloma drain outputs quickly decreased, with complete cessation of output by post-intervention day 2. Unfortunately, the output increased 6 days post-embolization with “black” output from the drainage catheter, presumed to represent embolic material. Eventually, repeat embolization was performed in a similar fashion to the index procedure, and no drain was left behind to prevent repeat inadvertent drainage of the embolic. Cholangiogram 2 weeks later demonstrated successful embolization. CT imaging follow-up 6 months later demonstrated no recurrent or persistent biliary leak.

## Case 2

**Fig. 2:**
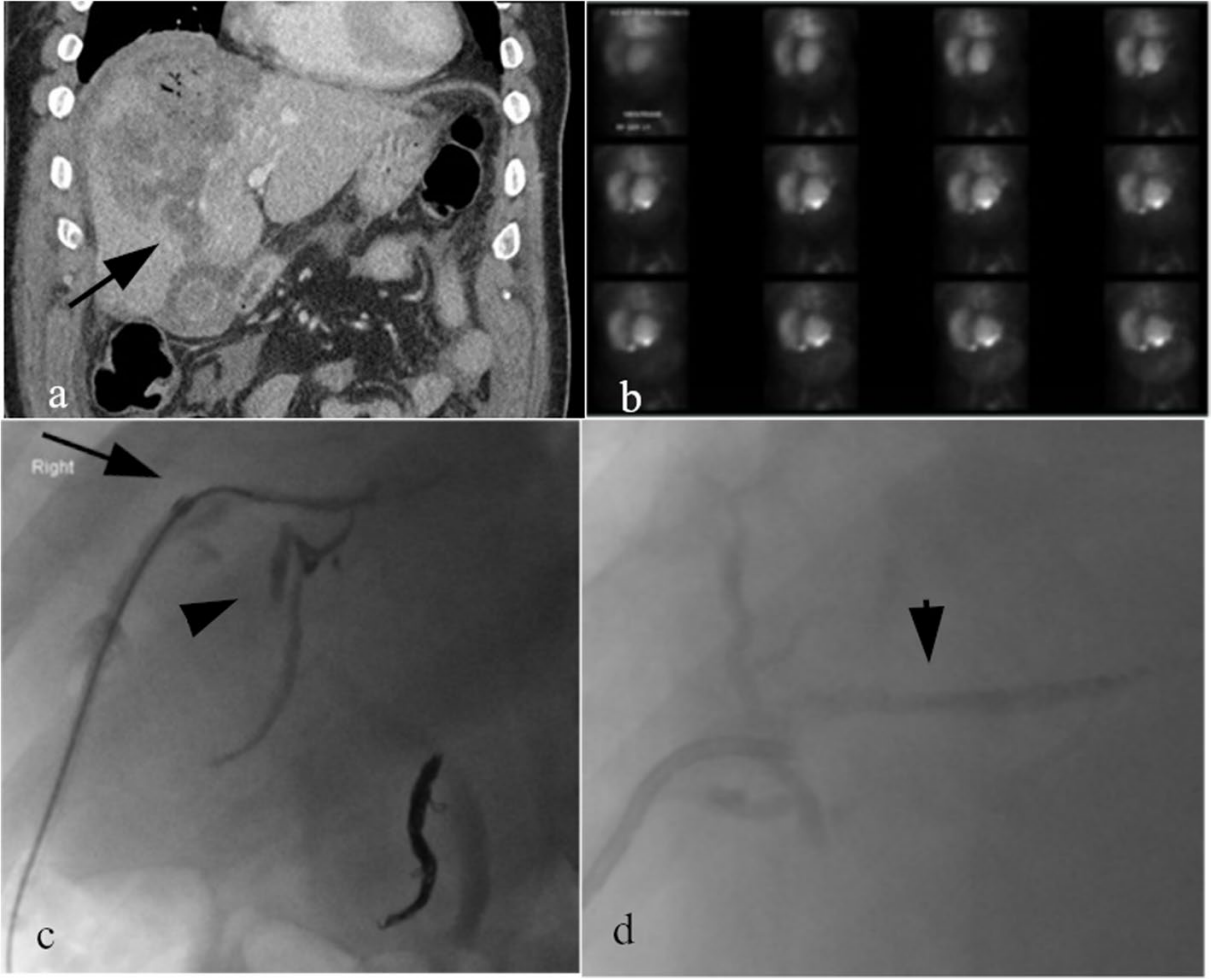
**a** High grade liver laceration seen on contrast enhanced CT. **b** HIDA scan with evidence of biliary leak. **c** Catheterization of hepatic duct (arrow) with contrast injection confirming site of leak (arrowhead). **d** Post embolization cast in site of biliary leak (arrow)

A 55-year-old man suffered multiorgan injury secondary to blunt force trauma from an E-Bike accident. Among his injuries was a high-grade liver laceration (Fig. 2a) which resulted in a biloma confirmed by HIDA (Fig. 2b). An ERCP was performed, and a CBD stent was placed for diversion, and the biloma was percutaneously drained. Despite these interventions, the drain continued to exhibit high bilious outputs. More aggressive endoscopic interventions were attempted; unfortunately, the leak was too peripheral to access via an endoscopic approach.

After 7 months of percutaneous drainage, the patient was brought to the IR suite for embolization. Using the combination of a sheath, an angled catheter, and a 2.4-F microcatheter, the leak was identified from the existing percutaneous access and the microsystem was used to selectively embolize multiple branches of the complex leak utilizing 0.3 cc of Obsidio embolic (Fig. 2c). An additional 0.2 cc of Obsidio embolic was then injected in a smooth and continuous fashion across the “cut surface” of the leak. A non-locking modified drainage catheter was placed as a safety drain and capped (Fig. 2d).

The patient returned 1 week later with follow-up CT demonstrating no evidence of biloma recurrence or bile leak. The capped drainage catheter was removed. Follow-up CT and HIDA scan 2 months later demonstrated no further evidence of bile leak or biloma recurrence.

## Case 3

**Fig. 3:**
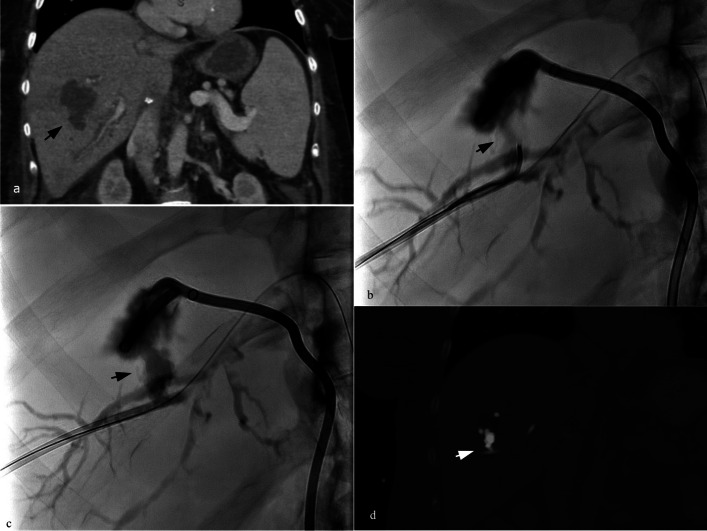
**a** Contrast enhanced CT abdomen demonstrate a 5.1 cm × 3.6 cm biloma seen within right hepatic lobe. **b** Catheterization of abnormal bile duct-biloma connection. **c** Post embolization image with Obsidio embolic within connection. **d** Coronal view of CT abdomen demonstrating post embolization changes

A 61-year-old female with a history of cholangiocarcinoma following liver transplantation was incidentally found to have a 5.1 cm × 3.6 cm biloma on contrast enhanced CT abdomen (Fig. 3a).

A multipurpose drain was placed with laboratory studies confirming a biloma. Additionally, contrast injection demonstrated a small connection between the biloma and a bile duct. Given persistent high output from biloma, a percutaneous transhepatic internal-external biliary drain was placed into the minimally dilated biliary tree to traverse and exclude the injured bile duct. The biloma drain output however remained elevated.

A cholangiogram was performed with visualization of the leak. The leak was catheterized from the right hepatic duct (Fig. 3b) and 0.6 cc of Obsidio was delivered in a slow and continuous fashion to embolize the biliary leak. The post-intervention cholangiogram showed no residual leak (Fig. 3c). A new 8.5-F internal–external right biliary drain was placed.

Over the subsequent two days, the biloma drain output precipitously dropped and the internal-external biliary drain was successfully capped. A repeat CT performed a month later showed near resolution of the biloma (Fig. 3d). She returned to IR two subsequent days later with cholangiogram demonstrating successful resolution of the biloma. Both drains were removed.

## Case 4

**Fig. 4:**
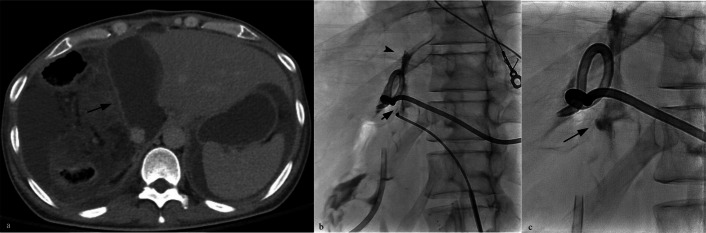
**a** Contrast enhanced CT abdomen demonstrate post hepatectomy biloma. **b** Catheterization of bile duct-biloma connection (arrow) with contrast injection filling biloma (arowhead). **c** Post embolization image with Obsidio embolic within connection as well as the biloma

A 48-year-old female previously underwent an extended right hepatectomy for HCC, presenting with bile leak identified on CT (Fig. 4a). A drain was placed within the biloma. The patient underwent ERCP with placement of a plastic biliary stent and a transhepatic internal–external biliary drain was placed into the mildly dilated biliary tree to exclude the area of leak. However there remained persistently high bilious outputs.

An over the wire cholangiogram was performed with catheterization of the fistula to the biloma (Fig. 4b). The biliary leak was embolized with injection of 0.5 cc of Obsidio delivered in a slow and continuous fashion (Fig. 4c). The post-intervention cholangiogram showed no evidence of residual leak. The internal–external biliary drain was replaced.

Over the subsequent days, the biloma drain output gradually decreased and the internal-external biliary drain was successfully capped. A repeat CT performed 2 weeks later showed near resolution of the biloma.

## Conclusion

These cases illustrate the use of Obsidio for embolization of refractory biliary leaks with durable clinical results. The mechanism of embolization by Obsidio includes cast formation as well as silicate particles inducing platelet adhesion/aggregation [2]. The ability to control the type and size of cast formed, as well as the conformable space-filling nature of the embolic material, are unique characteristics which may portend advantages during embolization in the biliary system. More research is needed to identify factors, such as anatomic (location and size of bile duct injury) as well as etiologic (traumatic versus post-surgical), which may be better suited for embolization with this material. These cases add to growing literature suggesting efficacy of this embolic agent in the management of biliary leaks [3, 4].
